# Morphological, Physiological and Transcriptomic Changes in Response to Water Deficit Stress in *Brassica napus* L.

**DOI:** 10.3390/ijms27177967

**Published:** 2026-09-07

**Authors:** Harsh Raman, Brett McVittie, Niharika Sharma, Maheswaran Rohan, Rosy Raman

**Affiliations:** 1 NSW Department of Primary Industries and Regional Development, Wagga Wagga Agricultural Institute, Wagga Wagga, NSW 2650, Australia; brett.mcvittie@dpird.nsw.gov.au (B.M.); maheswaran.rohan@flinders.edu.au (M.R.); rosy.raman@dpird.nsw.gov.au (R.R.); 2 NSW Department of Primary Industries and Regional Development, Orange Agricultural Institute, Orange, NSW 2800, Australia; niharika.sharma@dpird.nsw.gov.au

**Keywords:** *Brassica napus*, drought tolerance, trait plasticity, transcriptome analysis, differentially expressed genes

## Abstract

Yield losses due to water-deficit (WD) conditions, especially during the reproductive stages of plant development, pose a significant threat to global canola (*Brassica napus* L.) production. Therefore, it is critical to investigate traits contributing to improved productivity under increased WD conditions. Here we present phenotypic, physiological and transcriptomic changes in response to WD across contrasting canola accessions exhibiting variation in drought resistance-related traits. WD significantly reduced shoot biomass, plant height, harvest index, leaf water content, photosynthetic CO_2_ assimilation rate, intrinsic water-use efficiency and carbon isotope discrimination. WD caused 49 to 100% of the seed yield reduction: the minimum seed yield reduction (49.66%) was observed in a doubled-haploid (DH) line, 06-5101.137, while the maximum yield reduction (94.1 to 100%) occurred in the late-flowering DH lines (06.5101.088 and 06-5101.306). Seed yield showed a positive correlation (*r* = 0.29 to 0.95) with shoot biomass and harvest index, leaf water content, photosynthetic CO_2_ assimilation rate, intrinsic water use efficiency and carbon isotope discrimination. However, it showed negative correlations with days to flower, leaf specific weight, root length, root biomass (*r* = −0.04 to −0.79) across water treatments. The specific leaf transcriptome analysis of the two parental lines of DH population that exhibit variation for effective water use under well-watered and water-deficient conditions revealed different categories of differentially expressed genes (DEGs): WD-responsive DEGs in BC1329 parental line (1116) and BC9102 (1205) with 754 and 853 DEGs unique to BC1329 and BC9102, respectively, WD-responsive DEGs (906), genotype-dependent DEGs (8465) and genotype × treatment interaction DEGs (353). DEG annotations revealed that the WD-treatment-affected genes were involved in stress responses and growth and development. We further located 235 DEGs within the QTL regions underlying agronomic and physiological performance. Our study provides a conceptual framework for the morphological, physiological and molecular determinants involved in water-use efficiency. Seedlings’ traits with high heritability values, such as shoot biomass, leaf weight, leaf water content and Δ^13^C, serve as proxies for trait-based selection for improved seed yield under both water-limited and non-water-limited conditions.

## 1. Introduction

Climate change models suggest that drought (water-deficit, WD) events will likely increase and disproportionately impact crop yields and food security within the next 20 years in various parts of the world [1,2]. During the last three decades, drought alone caused more than $30 billion in yield losses [3]. To mitigate the negative effects of drought stress, increase yield and reduce the yield gap in water-limited environments, genomic improvement of effective water use and drought resistance traits is one of the promising strategies. However, developing drought-resilient crops relies on a detailed understanding of trait variation, genetic factors and their molecular regulatory networks underpinning sustainable crop production.

In nature, plants acclimatise to WD by adjusting growth and fine-tuning metabolism by deploying different strategies. For example, they escape from drought by shortening their life cycle [4,5] and tolerate WD via the accumulation of metabolites and proteins, protecting cell integrity caused by low water potential [6]. Plants can also avoid WD stress by maintaining tissue hydration, reducing water loss due to transpiration (water use efficiency, WUE) and enhancing water uptake so that they bypass the detrimental effects of water-deficit conditions [7].

Genetic variation in drought avoidance traits such as transpiration efficiency (TE, total biomass produced per unit of transpired water); CO_2_ assimilation, *A_g_*; stomatal conductance, *g_sw_*; intrinsic water use efficiency (*i*WUE, the ratio between *A_g_* and *g_sw_*: *A_g_/g_sw_*) and its proxy trait, e.g., leaf carbon isotope discrimination (Δ^13^C) or carbon isotope composition (δ^13^C, ^13^C/^12^C); and root traits such as root pulling force, root length and root biomass [8,9,10,11] are shown to contribute to yield improvement. However, some traits related to WUE at the single-leaf (TE) and the whole-plant level, such as leaf water content, specific leaf weight and aerial shoot biomass, do not consistently correlate with grain yield across environments and plant species [7,12,13,14]. Therefore, breeding crops for high TE has been debated, with some arguing that effective water use (EWU) is a better predictor of achieving a high yield than reduced water usage [15,16]. The reduced water use attributed to low stomatal conductance is also implicated in slower plant growth, resulting in lower yield under terminal drought conditions [8]. Therefore, it remains to be established which phenological and physiological traits are relevant for making genetic gains in breeding programs to enhance crop productivity in WD conditions.

Canola (rapeseed, *Brassica napus* L.) is the second-largest oilseed crop in the world and is cultivated across continents in arid and semiarid temperate regions. Different varieties exhibit variations in drought avoidance and drought escape traits, reflecting adaptation to WD environments [17,18]. Quantitative trait loci (QTL) for variation in Δ^13^C, root pulling force, root biomass and drought tolerance-related traits have been identified in canola [17,18,19,20,21,22,23]. Interestingly, some QTL for Δ^13^C, days to flower, plant height and seed yield are collocated near the flowering time homologues such as *FLOWERING LOCUS T* and *FLOWERING LOCUS C*, which account for significant (~40%) trait variation across environments [17,18,21,22]. These studies indicate a possible trade-off between early flowering and WUE in canola. Recently, one candidate gene in *B. napus* has been shown to confer drought tolerance at the seedling stage [24]. In *Arabidopsis thaliana*, several genes have also been shown to improve drought tolerance in several crop plants, including barley, wheat, canola, cotton, maize, peanut, rice, soybean and tomato [8,25,26]. However, the significance of those genes in response to WD and alleviating WD yield losses in canola is unknown.

In earlier studies, canola parental lines, BC1329 and BC9102, revealed a genetically determined variation in seed yield, TE and WUE-related traits [17,22,27]. Next-generation sequencing approaches, such as RNA sequencing, have been utilised to identify the putative genes and molecular mechanisms of plant responses to drought stress in plants [28]. Herein, we investigated (i) morphological, physiological and productivity trait variation among 24 *B. napus* accessions in contrasting water regimes: well-watered (WW, control) and water-deficit (WD) conditions and (ii) the transcriptomic dynamics in two parental lines of a doubled-haploid population of *B. napus* derived from BC1329 (maternal) and BC9102 (paternal) lines under WD. Our results on phenotypic, physiological and transcriptomic details provide insights into transcriptional reprogramming, pathways, and regulatory networks associated with WD, drought tolerance, productivity-related traits and putative gene targets for genetic intervention to cope with water deficits. These findings can be further verified for the sustainable and profitable production of *B. napus* varieties under moderate WD conditions.

## 2. Results

### 2.1. Genetic Variation in Different Traits

**Figure 1:**
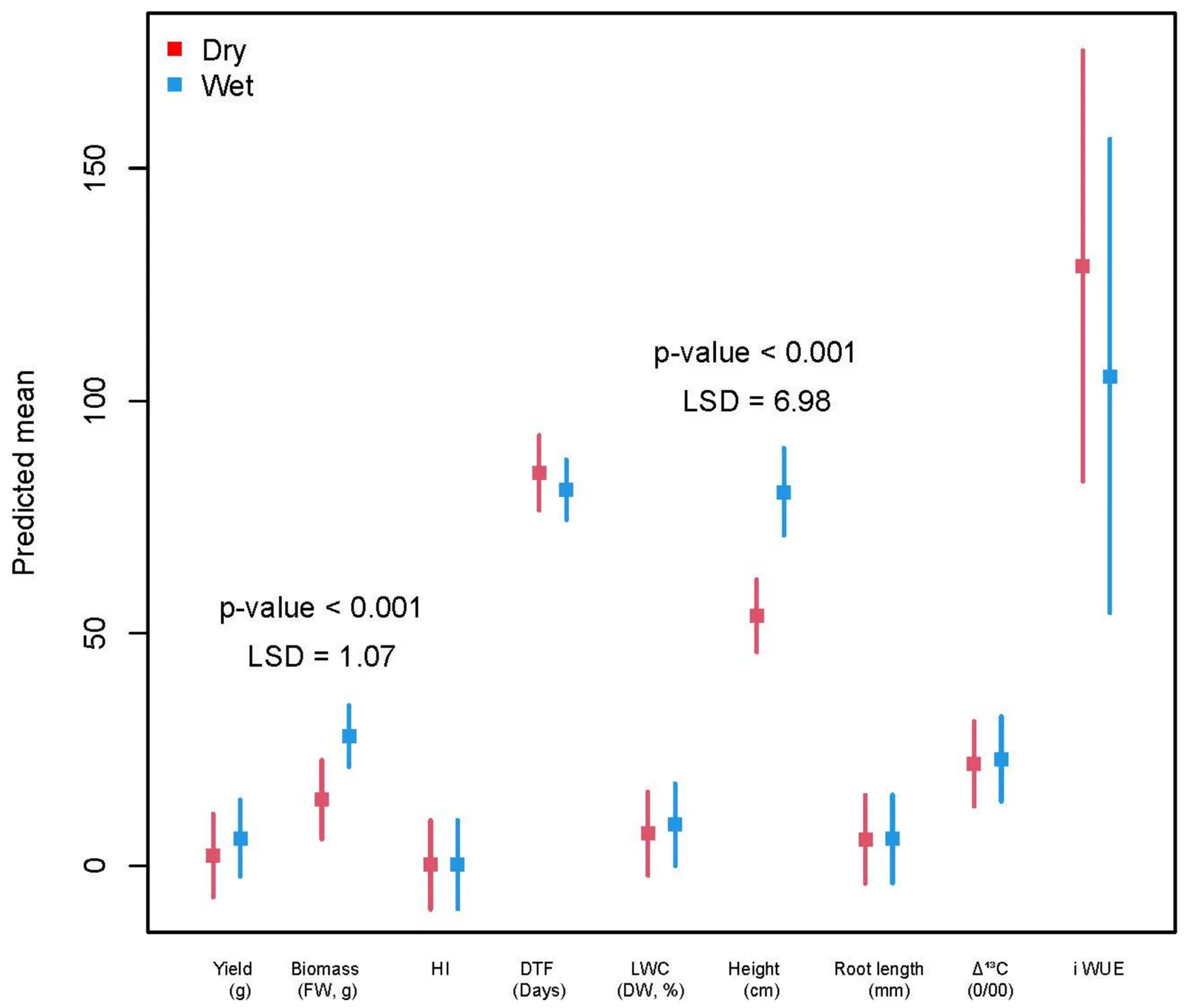
Changes in morphological and physiological phenotypes: seed yield, shoot biomass, harvest index (HI), days to flower (DTF), leaf water content (LWC), plant height, root length, Δ^13^C and intrinsic water use efficiency (*i*WUE) in 24 accessions of *B. napus* subjected to water treatments (WW: well-watered and WD: water deficit). LSD values to compare accessions at *p* < 0.001 (1.07) and treatment groups (6.98) are given. The data represent the predicted means of six independent replications (*n* = 6).

At the seedling stage (short-term; dry-down experiment I, Table 1), we measured the shoot biomass to study the genetic variation and response of accessions to WD. Fisher’s Least Significant Difference (LSD) values to compare treatment, and genotypic effects are presented (Figure 1, Table S2). We observed high accuracy values, similar to *H*^2^ (0.94 to 0.97), for shoot biomass across water treatments (Table 2). There were significant differences in shoot biomass accumulation within and between treatments; trait means of all 24 accessions were higher in the WW compared to the WD treatment, as expected (Table S2). In the WW treatment, an Australian winter-type hybrid cultivar, Hyola970CL, had the maximum shoot biomass (46.40 g), while the fodder-type turnip cultivar, NZ Sensation, had 3.375× lower shoot biomass (13.84 g), reflecting the existence of substantial trait variation among accessions (Table S3a). As expected, both shoot biomass expressed at fresh and dry weight bases showed a high correlation (*R*^2^: 0.83, Figure S1). The NZ Sensation showed the least response to WD and lost only 12.57% of shoot biomass, while the BC1329 maternal line of the 5101-DH population showed the highest sensitivity to WD and lost 56.60% of shoot biomass when compared to shoot biomass in the WW treatment (Table S3).

In experiment II (Table 1), we observed significant variation in traits related to plant development: flowering time, plant height and shoot and root biomass at physiological maturity; physiology (Δ^13^, leaf weight/leaf disc weight and leaf water content); and productivity parameters (seed yield and harvest index), in both WW and WD regimes (Figure 1, Tables S2 and S3). There were high *H*^2^ values (0.85 to 0.98) for most of the traits under both WW and WD conditions, except for *A* and *g_sw_* measures and root length, where moderate *H*^2^ values (0.40–0.78) were observed (Table 2).

BC9102 exhibited high predicted mean values for seed yield, harvest index and Δ^13^C, and lower mean values for shoot biomass, days to flower, plant height, root length and root weight than BC1329. These results are consistent with our previous field and rainout shelter-based studies [17,22]. BC9102 had the maximum seed yield (12.41 g/plant), whereas the 06.5101.009 DH line had the minimum yield (up to 0.41 g/plant) under WW conditions. Likewise, variation in seed yield was also observed in WD treatment; CB-Tefler, an Australian cultivar bred for a shorter season under rainfed conditions, had the highest seed yield (4.61 g/plant), followed by BC9102 (4.57 g/plant), whereas Hyola970CL, NZ Sensation and the 06.5101.306 DH lines did not yield at all, as their vernalisation requirement may have been met late in the season and they did not fill their seeds possibly due to the onset of high temperatures in a glasshouse. It is mentioned that evaporative cooler-based air-conditioning is not efficient for maintaining set temperatures in the late spring (November–December) months in Australia when temperatures exceed more than 35° to 40 °C. Therefore, we terminated the experiment, and data of Hyola970CL and NZ Sensation were not considered for correlation analysis of yield and HI.

### 2.2. Phenotypic Correlations Between Morphological, Physiological and Productivity Traits

**Figure 2:**
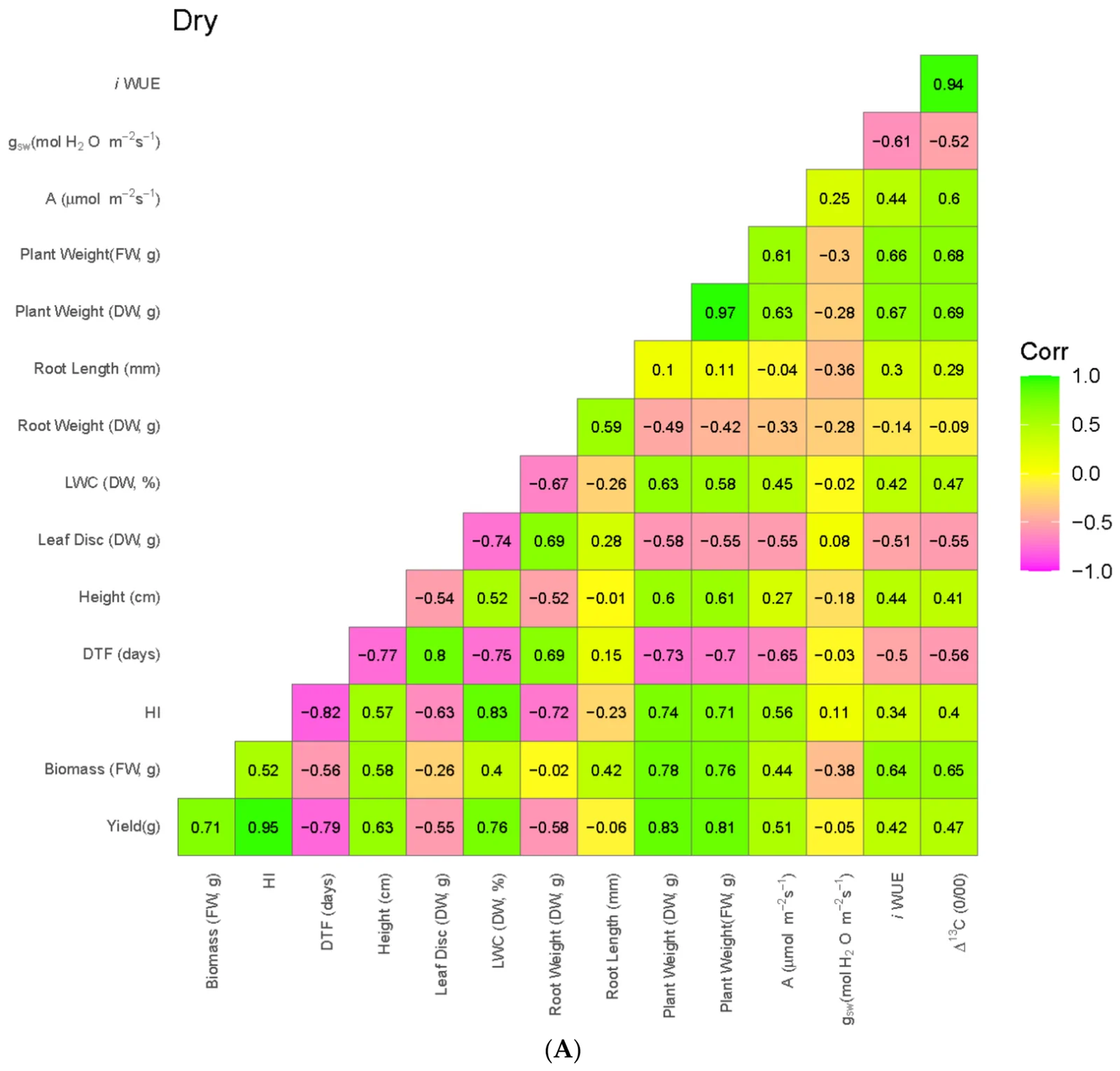

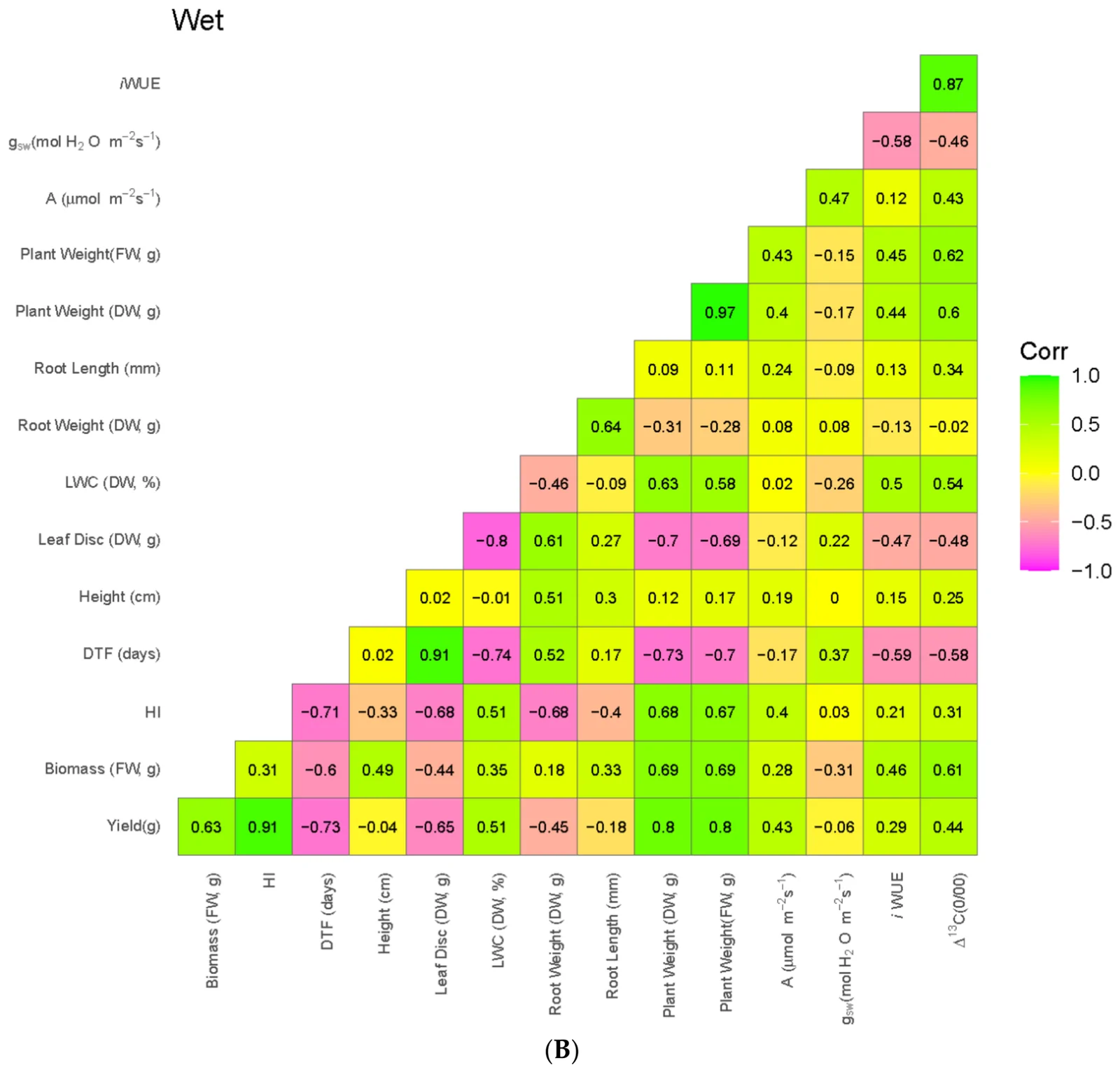
Trait correlations of 24 *B. napus* accessions, evaluated under WW (**A**) and WD (**B**) conditions in a glasshouse. Yield (seed yield, g/plant), HI: harvest index, DTF: days to flower, Height (plant height), Leaf Disc (DW, dry weight), LWC: leaf water content, Root weight (DW, dry weight), Root length, Plant weight (expressed on dry weight, DW and fresh weight, FW basis), *A*: CO_2_ assimilation rate; g_sw_: stomatal conductance, *i*WUE: intrinsic transpiration efficiency (*A_g_*/*g_sw_*) and Δ^13^C: carbon isotope discrimination, ‰). Empirical best linear unbiased predictions (E-BLUPs) of accessions were estimated from a separate analysis of each trait and plotted. Pearson’s correlation coefficient between traits are presented in square boxes.

We further performed correlation analysis to unveil the relationships between plant development, productivity and physiological traits. Shoot biomass (fresh weight at the seedling stage, experiment 1), HI, LWC, plant/shoot biomass (expressed on a dry and fresh weight basis at physiological maturity), *A*, *i*WUE and Δ^13^C showed positive correlations (*r* = 0.29 to 0.95) with seed yield in both WW and WD conditions (Figure 2A,B). In contrast, days to flower, leaf-specific weight (LSW, leaf disc weight), root length, root biomass and *g_sw_* were negatively correlated with seed yield (*r* = −0.04 to −0.79). Plant height showed an invariable relationship across water treatments; it showed a positive correlation (*r* = 0.63) with seed yield in the WD (DRY) treatment, whereas it showed a negative correlation (*r* = −0.04) in the WW (WET) treatment, likely due to the non-determinate growth habit of *B. napus* (Figure 2A,B). Lines having low seed yield (06-5101.009, 06-5101.018, 06-5101.088, 06-5101.226 and 06-5101.306) were later in flowering time than earlier ones (Table S3b). Flowering time was positively correlated with LSW across water treatments (*r* = 0.80 to 0.91). Lines with higher Δ^13^C had higher LWC and revealed an inverse relationship with LSW (Figure 2). In general, the correlation between traits was stronger in the DRY (WD) treatment compared to the WET (WW) treatment.

### 2.3. Response of WD on Agronomic and Productivity Traits

To determine how different accessions respond to DRY (WD) treatment, we compared the responses of all 24 accessions to water deficit, as a deviation in BLUPS between WW and WD treatments (experiment II). The WD caused a significant reduction in all traits measured, except for days to flower (Tables S2 and S3). It significantly reduced fresh biomass (up to 56.6%), LWC (up to 36%), Δ^13^C (4.5%), plant height (up to 81.9%), root biomass (78%) and seed yield and HI (up to 100%); see Table S3b. WD stress reduced the seed yield of canola by at least 49.65% of the yield (WW); a DH line, 06-5101.137, showed the minimum seed yield reduction (49.66%), while the late-flowering lines (06.5101.088, 06-5101.306) had the maximum reduction (94.1 to 100%). There was a linear relationship between seed yield across WW and WD conditions (*R*^2^ = 0.89, Figure S2). However, some genotypes changed their ranks (Figure S3). There was no significant treatment effect on the flowering time trait; the majority of accessions exhibited late flowering under WD conditions (Table S3b), suggesting that WD does not univocally accelerate flowering in *B. napus* due to water stress.

Flowering time showed high heritability values across treatment groups, indicating that it is a stable trait, irrespective of water treatment. The majority of varieties improved HI in WW treatment; however, there was no change in HI among early-flowering varieties 06.5101.014, 06.5101.026 and 06.5101.137 under the WD treatment (Table S3b). We observed that accessions most sensitive to WD were darker green and waxier than well-watered plants, consistent with the earlier observation [21]. WD conditions induced a waxy phenotype among accessions, highly pronounced in Hyola970CL. Further research is required to determine the role of leaf waxiness in transpiration/water use efficiency and water productivity.

### 2.4. Response of WD on Physiological Traits

Previous studies showed that high *A_g_* and low *g_sw_* improve the *i*WUE and seed yield. In this study, we found that accessions grown under the WW and WD did not show any statistically significant genotypic difference in *A* and g_sw_ rates. Higher values for *A* and *g_sw_* were observed (up to 6.17 µmol CO_2_ and 0.05 mol H_2_O m^−2^s^−1^, respectively) in WW compared to the WD conditions (Table S3c). This may have occurred due to the intricate nature of LICOR measurements and physiological interplay between gas exchange dynamics in the test plants and vapour pressure deficit conditions occurring during the experimentation. There was a 17.64% to 61.29% reduction in *A* (06.5101.501) and 0.40% to 54.82% *g_sw_* (06.5101.088) in WD conditions (Table S3c).

Among accessions, there were significant differences in variation in LSW and Δ^13^C signatures across treatments. WD reduced the LWC by 11.72% in CB-Telfer to 36.03% in the 06.5101.157 DH line. There was no reduction in LSW among the late-flowering 06.5101.009, 06.5101.063, 06.5101.088, 06.5101.306 and Hyola970CL lines, whereas early-flowering lines (06.5101.14, 06.5101.26, 06.5101.137, 06.5101.157, 06.5101.643, CB-Telfer, SturtTT, RocketCL and BC9102) showed up to 25% reduction in LSW (Table S3). Both traits, flowering time and LSW, showed a high correlation (0.80 to 0.91%) under WW and WD conditions. WD reduced the Δ^13^C by 2.53‰ in CB-Telfer to 6.34‰ in Hyola970CL. These results suggest that genetic variation in several physiological traits, such as LSW, LWC and Δ^13^C under WD conditions, exists among the tested canola accessions.

### 2.5. Transcriptomic Response of WD on Two Parental Lines

To delineate a comprehensive repertoire of transcriptional reprogramming, pathways and regulatory networks associated with water deficit, we performed RNA sequencing of 12 samples (two genotypes: BC1329-P1 and BC9102-P2; ×2 conditions: control—well-watered (saturated at 100% field capacity, WW, wet) and treated—water deficit (watered at 50% field capacity, WD, dry); ×3 biological replicates). RNA sequencing generated a total of 33.75 Gb of data across 12 libraries, corresponding to 20.7–39.6 million reads per sample. Between 69.7% and 81.1% of reads mapped to the *B. napus* reference genome, with 64.8–75.2% mapping uniquely. The proportion of uniquely mapped reads was consistent across all samples, with no obvious differences between genotypes or watering treatments. Approximately 4.9–7.2% of total reads mapped to genomic regions lacking annotated features, likely reflecting unannotated transcripts, non-coding RNAs, or incomplete gene annotation. The summary of sequence data generated and reads mapped to the reference genome is presented in Table S4. These results indicate good overall alignment quality and support the reliability of downstream differential expression analyses. The dataset was further explored to investigate relationships between samples, and Figure S4 shows that the distances between samples correspond to the biological coefficient of variation of the samples.

### 2.6. Differential Gene Expression Analysis

**Figure 3:**
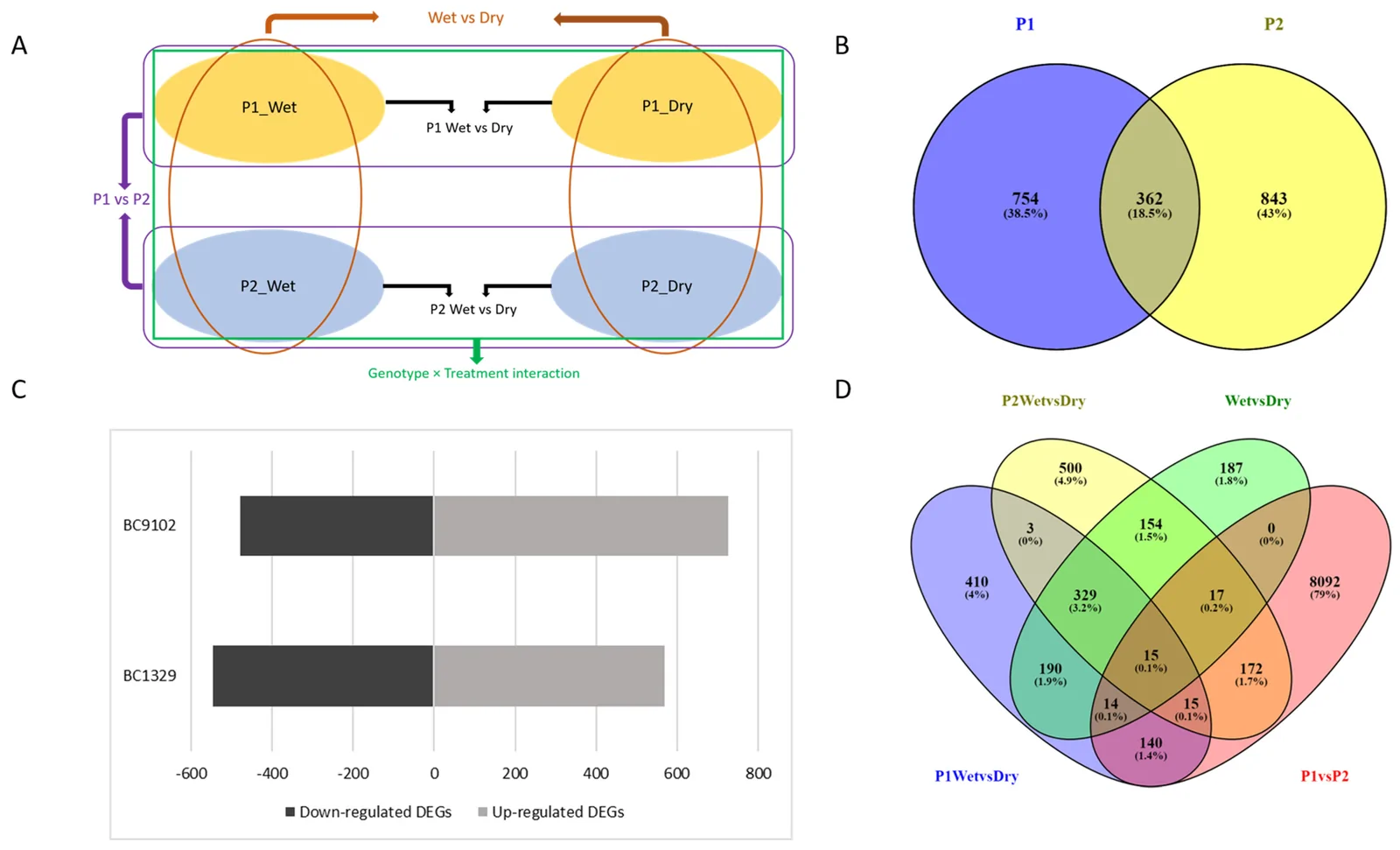
(**A**) Graphical representation of the experimental design, including parental lines and water treatment information for differential gene expression comparisons. Cartoon showing the four experimental groups (P1_Wet, P1_Dry, P2_Wet and P2_Dry) that were compared (shown in different colours) to test each contrast, resulting in a list of DEGs. (i) Water-deficit-responsive DEGs in P1 (BC1329) = P1_Wet vs. P1_Dry comparison (black colour) and P2 (BC9102) = P2_Wet vs. P2_Dry comparison (black colour); (ii) genotype-dependent DEGs = (P1_Wet and P1_Dry) vs. (P2_Wet and P2_Dry) comparison (purple colour); (iii) treatment-dependent DEGs = (P1_Dry and P2_Dry) vs. (P1_Wet and P2_Wet) comparison (brown colour); and (iv) DEGs with genotype × treatment effect = (P1_Wet vs. P1_Dry) vs. (P2_Wet vs. P2_Dry). (**C**): Bar graph showing the number of upregulated and downregulated WD-responsive DEGs in two parental lines, BC1329 (P1) and BC9102 (P2). (**B**): Venn diagram representing common and unique water-deficit-responsive DEGs in BC1329 (P1) and BC9102 (P2). Unique genes are represented in individual blue and yellow circles, whereas the common genes are represented as the middle-integrated ones. (**D**): Venn diagram showing common and unique DEGs in four comparisons: P1 Wet vs. Dry, P2 Wet vs. Dry, P1 vs. P2 and Wet vs. Dry.

Differential expression analysis was performed across four experimental groups (P1_wet/control–BC1329_WW/wet; P1_dry/treated–BC1329_WD/dry; P2_wet/control–BC9102_WW/wet; P2_dry/treated–BC9102_WD/dry) using linear models, yielding meaningful comparisons. Figure 3A shows the comparisons and the resultant four categories of differentially expressed genes (DEGs): (i) WD-responsive genes in BC1329 (P1 Wet vs. Dry) and BC9102 (P2 Wet vs. Dry); (ii) genes exhibiting genotype-dependent expression differences (P1 vs. P2); (iii) genes showing water-deficit and non-water-deficit expression differences (Wet vs. Dry); and (iv) genes with genotype × treatment interaction effect.

#### 2.6.1. Water-Deficit-Responsive Genes in Two Parental Lines

Both parental lines (BC1329 and BC9102) exhibited a significant number of DEGs in response to WD/dry conditions. BC1329 (P1, less effective water use, higher seed yield) had 1116 WD-responsive DEGs, of which 570 genes were upregulated, whereas 546 genes were downregulated (in P1 Wet vs. Dry comparison) (Figure 3C, Table S5). Similarly, WD resulted in 1205 DEGs in BC9102 (P2, more effective water use), where 726 and 479 genes were upregulated and downregulated, respectively (in P2 Wet vs. Dry comparison) (Figure 3C, Table S6). Both parental lines had 362 common WD-responsive DEGs, and 754 and 843 genes were unique WD-responsive DEGs in BC1329 (P1) and BC9102 (P2), respectively (Figure 3B). Comparison of DEGs in four categories further revealed substantial variation in gene expression patterns among contrasts. A total of 15 genes were consistently differentially expressed across all four comparisons, suggesting a core set of genes associated with both genotype and treatment effects (Figure 3D).

**Figure 4:**
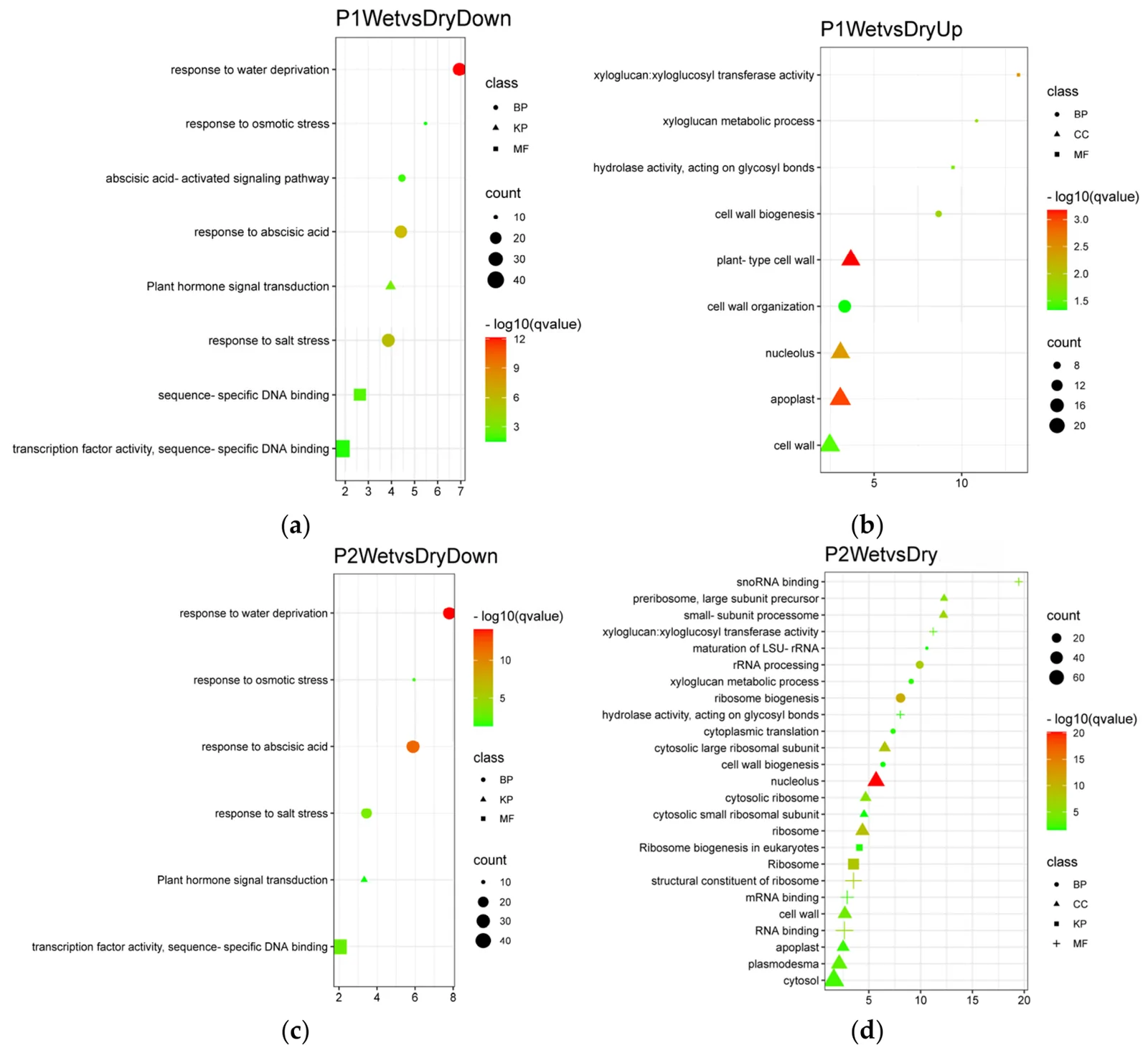
Bubble plot showing enriched gene categories in (**a**) downregulated WD-responsive DEGs in P1 and (**b**) upregulated WD-responsive DEGs in P1. (**c**) Downregulated WD-responsive DEGs in P2 and (**d**) upregulated WD-responsive DEGs in P2.

Gene Ontology (GO) enrichment and Kyoto Encyclopedia of Genes and Genomes (KEGG) pathway analyses (Figure 4a; Table S7) revealed that the downregulated genes in BC1329 (P1 Wet vs. Dry comparison) were mainly involved in response to water deprivation, response to abscisic acid, response to salt stress, and response to osmotic stress. In contrast, the upregulated DEGs in this category were implicated in cell wall biogenesis, cell wall organisation and xyloglucan metabolic processes (Figure 4b). The KEGG pathway analysis revealed that both up- and downregulated genes were significantly associated with the plant hormone signal transduction pathway. Downregulated genes in the BC9102 (P2 Wet vs. Dry comparison) exhibited the same significant GO terms as mentioned above (Figure 4c, Table S8). On the other hand, most of the upregulated genes in the P2 Wet vs. Dry category were involved in ribosome biogenesis, rRNA processing, cytoplasmic translation and cell wall biogenesis. Ribosome biogenesis and DNA replication were the two significant KEGG pathway terms associated with P2 Wet vs. Dry DEGs (Figure 4d).

Interestingly, all 362 common WD-responsive genes showed the same direction of expression change in both parental lines (positive or negative fold changes in both P1 Wet vs. Dry and P2 Wet vs. Dry comparisons), suggesting a similar primary response mechanism to WD in P1 and P2. Twenty-six genes exhibited at least or more than two units of difference in the fold change values in both P1 Wet vs. Dry and P2 Wet vs. Dry comparisons, and are mainly involved in plant growth and development, cellular biogenesis, abscisic acid-mediated signalling and water deprivation. Of 26 genes, 13 showed higher fold changes in the BC1329 parental line (P1, less effective water use) and were associated with abscisic acid-mediated signalling and water deprivation. The remaining 13 genes with higher fold changes in BC9102 (P2, more effective water use) were involved in shoot development, cell differentiation, osmotic stress and hormonal response. GO enrichment analysis further revealed that the common WD-responsive DEGs were mainly involved in response to water deprivation, response to abscisic acid, response to osmotic stress and response to salt stress.

**Figure 5:**
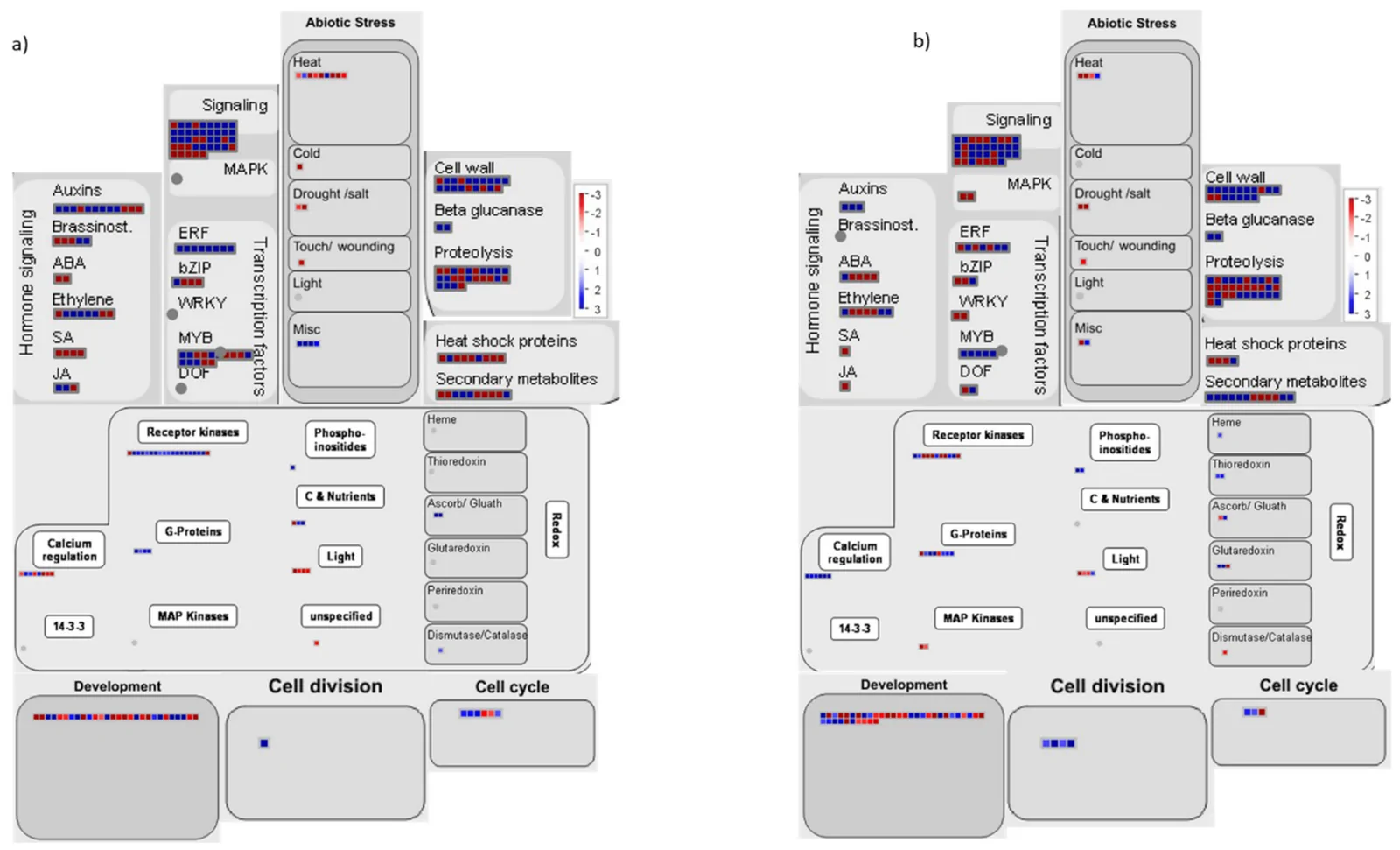
MapMan pathway views of water-deficit-responsive genes unique to P1 and P2. (**a**) Unique WD-responsive genes in BC1329 (P1), and (**b**) unique WD-responsive genes in BC9102 (P2). Blue and red colours indicate up- and downregulation of DEGs, respectively, and colour intensity reflects the magnitude of change.

In the P1 Wet vs. Dry comparison, 96 DEGs were identified with roles in response to water deprivation, whereas the P2 Wet vs. Dry comparison revealed 90 genes in this category; most genes were downregulated in both comparisons (76 and 72, respectively). This suggests that these common WD-responsive genes in two parental lines provide signal transduction, hormone-mediated signalling and stress response, and are associated with the execution of key functions under water stress. In contrast, the WD-responsive genes unique to BC1329 (P1, more effective water use) and BC9102 (P2, less effective water use) showed some expression differences across functional categories, as shown in Figure 5. Hormone signalling (auxin, brassinosteroids, abscisic acid, jasmonic acid), heat shock proteins, secondary metabolites, transcription factors (ERFs, WRKY, MYB and DOFs) and redox state show striking expression differences among the unique WD-responsive genes in the two parental lines. In P1, unique DEGs were associated with response to water deprivation, hypoxia, salt stress, abscisic acid, jasmonic acid and glucose starvation. Significant GO terms with unique DEGs in P2 included rRNA processing, ribosome biogenesis and LSU-rRNA maturation.

#### 2.6.2. Genes Exhibiting Genotype-Dependent Expression Differences

**Figure 6:**
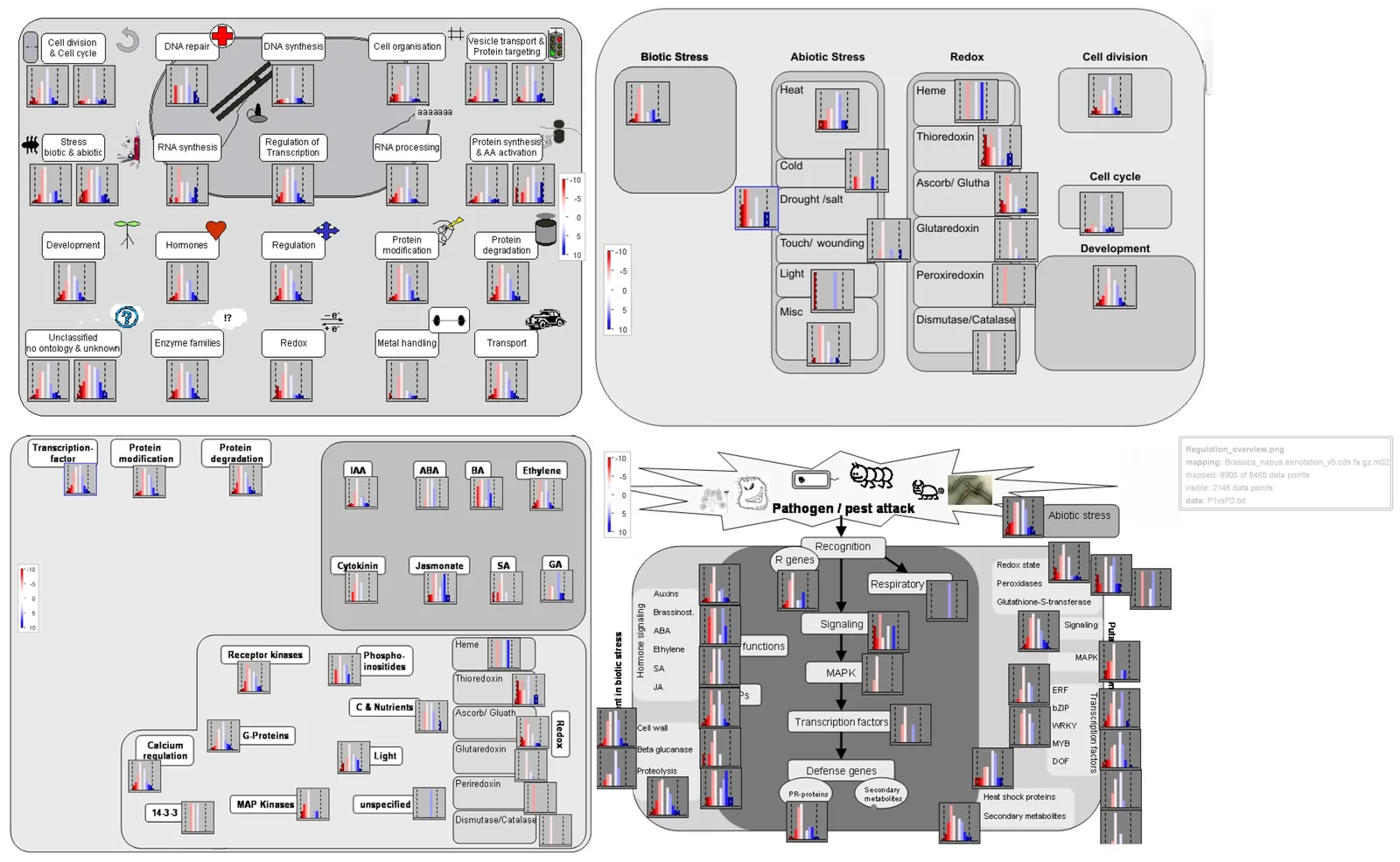
MapMan pathway views of DEGs exhibiting genotype-dependent expression differences. Blue and red colours indicate up- and down-regulation of DEGs, respectively, and colour intensity reflects the magnitude of change.

To investigate genotype-dependent expression differences, we performed comparative transcriptome analysis of both wet and dry samples in the two parental lines and identified 8465 DEGs with genotype-dependent expression changes (Figure 6, Table S9). This category had the highest number of genes among the comparisons. In this category of DEGs, 4530 genes (with negative FC values) depicted higher expression in BC9102 (P2 control and treated) samples than in BC1329 (P1 control and treated) samples. In contrast, 3935 genes (with positive FC values) showed higher expression in BC1329 (P1) samples than in BC9102 (P2) samples.

DEGs with higher expression in P2 samples were mainly involved in defence responses, whereas those with higher expression in P1 were mainly associated with DNA replication and cell division (Table S10). MapMan analysis of these DEGs indicated that a large fraction of genes with higher expression in P2 samples were involved in stress response, RNA synthesis, redox, hormonal response (cytokinin, salicylic acid) and signalling. In comparison, most of the DEGs with higher expression in P1 samples were involved in DNA synthesis and repair, RNA processing, protein synthesis, response to GA and stress (Figure 6).

Thus, the unique WD-responsive DEGs in BC1329 and BC9102, and the DEGs exhibiting genotype-dependent expression changes, indicate that the two parental lines exhibit different mechanisms to combat water stress.

#### 2.6.3. Genes Exhibiting Treatment-Dependent Expression Differences

This category contains 906 DEGs, among which WD treatment affected gene expression (Figure 6, Table S11). MapMan analysis showed that the downregulated genes in this category were secondary metabolites and heat shock proteins and were mainly involved in stress, transport, protein degradation and modification, ABA and ethylene signalling. In contrast, the upregulated genes were implicated in protein synthesis and regulation, as well as in auxin, jasmonate and GA signalling. GO enrichment analysis exhibited that these DEGs were mainly associated with response to water deprivation, response to abscisic acid, response to salt stress, response to osmotic stress, hyperosmotic salinity response, xyloglucan metabolic process, response to brassinosteroid, cell wall biogenesis, rRNA processing and response to salicylic acid. This category of DEGs represents common water-stress responses and was not further explored.

#### 2.6.4. Genes Exhibiting Genotype × Treatment Interaction Effect

The genotype × treatment interaction analysis revealed 353 DEGs, showing that WD responses differed markedly between the two genotypes, with interaction DEGs enriched for stress signalling, defence, detoxification and metabolic regulation pathways (Table S12). Key genes included WRKY (WRKY18, WRKY33, WRKY40) and ERF (ERF1, ERF2) transcription factors, JAZ regulators of jasmonate signalling, multiple glutathione S-transferases, lipoxygenases, ABC transporters and several pathogenesis-related proteins such as chitinases, β-1,3-glucanases, thaumatins and PR proteins. Genes involved in oxidative stress responses, sulphur assimilation (APR1, APR3, LSU1, LSU2, SDI1), secondary metabolism and transport processes also showed significant interaction effects. Collectively, these results indicate that the two canola genotypes employ distinct molecular strategies under water deficit, differing in their activation of defence, hormone signalling, redox regulation and nutrient metabolism pathways.

### 2.7. Identification of DEGs Within QTL Intervals

To identify potential candidate genes, we mined DEGs that map within 100 kb up- and downstream of the physical map positions of significantly associated markers underlying QTL for main effects: corresponding to genotypic (G) effects and QTL (Q)  ×  environment (E) interactions; corresponding to G  ×  E effects for variation in leaf Δ^13^C, flowering time, chlorophyll content (SPAD), early vigour (normalised vegetative difference index), plant height and seed yield in the DH population derived from a cross between BC1329 and BC9102 in field and rainout shelter environments under WW and WD conditions [17,22]. In our previous study, we localised candidate genes/DEG for leaf Δ^13^C [17]. In this study, we obtained 235 DEGs in the vicinity of QTL regions associated with drought escape (days to flower, plant height), drought avoidance (NDVI, Δ^13^C, SPAD) and seed yield on A01, A02, A03, A05, A07, A08, A09, A10, C02, C02_random, C03, C05, C06, C07, C08, C09 and C09_random chromosomes of *B. napus* (Table S13). Multi-trait QTL were mapped to similar genomic regions, such as for Δ^13^C, DTF, PH and SY on chromosome A09 and Δ^13^C and DTF on C09 (Table S13). A total of 212 DEGs were located within 100 kb of significant DArTseq-SNPs associations; of these, 25 DEGs were within 10 kb (Table S13).

For flowering time, we localised 90 DEG on chromosomes A01, A05, A08, A09, A10, C02, C06, C08 and C09. *BnaC02g43470D* (*MADS AFFECTING FLOWERING 3*, *MAF3* [29]) was differentially expressed (−3.398 folds) between parental lines of the DH population, revealing genotype-dependent downregulated expression (Table S13). *MAF3* was localised within 4.53 kb of the significant SNP associated with flowering time under three environmental conditions [17,22]. The other DEGs of interest were BnaA05g32570D (Short-chain dehydrogenase), *BnaA08g18990D* (Galactose oxidase), *BnaC02g45160D* (dehydrin) and *BnaCnng04580D*, which mapped closely to *TEMPRANILLO* 1 (*TEM1*), a transcriptional repressor of *FT* that participates in multiple flowering pathways and negatively regulates the juvenile-to-adult transition and the flowering transition [30]. QTL regions underlying chlorophyll content, measured with a SPAD meter on chromosomes A01, A02, A03, A09 and A10, harboured 48 DEGs within 100 kb, including Nuclear Factor Y subunit A1, Cytochrome p450 and Autoinhibited Ca^2+^ATPase whereas *BnaA03g04040D* (a transcription factor; HAP2A; EMBRYO DEFECTIVE 2220 showed 10.60-fold upregulated expression and mapped within 1 kb from a significant SNP associated with SPAD. (Table S13). For NDVI, three DEGs involved in protein autophosphorylation (*BnaA01g04670D*), isoleucine biosynthetic process (*BnaC06g15510D*), gametogenesis, control of mitotic progression and cell differentiation during the entire life cycle (*BnaC06g15530D*) were upregulated between parental lines. *BnaC06g15420D,* involved in circadian rhythm, seed germination and cell differentiation, was downregulated in P1 when subjected to WD (Table S13).

For leaf Δ^13^C QTL, we prirotised 21 DEGs on chromosomes A02, A07, A08, A09, C06 and C09 within 100 kb from SNP associations (Table S13). Approximately 50% of them (12/21) showed more than a three-fold difference in gene expression between parental lines/treatments; DEG *BnaA02g15890D* and *BnaA07g11440D*, encoding Auxin response SAUR, *BnaA07g11350D*, *BnaA09g42040D* (cation-transporting P-type ATPase, HMA1, implicated in growth) [31] and *BnaC09g46950D,* which encodes a basic helix-loop-helix DNA-binding superfamily protein (HECATE 3) implicated in phytohormone control [32], showed up to 9.72-fold differences in DEG (Table S13). For plant height, we localised 54 DEGs on chromosomes A08, A09, C03, C07 and C09, adjacent to significant SNP markers (Table S13). Of them, one DEG, *BnaC07g23900D*, was located within the 10 kb region.

For seed yield, we identified 38 DEGs within 100 kb from 11 QTL regions including genes *BnaA01g08090D* (MYB-CC type transcription factor) on A01; *BnaA08g13480D* (Acid phosphatase), *BnaA08g13490D* (MATE), *BnaA08g13540D* (Concanavalin A-like lectin protein kinase) on A08; *BnaA09g42040D* (Cation-transporting P-type ATPase) on A09; *BnaA10g04040D* (Fatty acid desaturase type 1), *BnaA10g03830D* (Inositol polyphosphate-related phosphatase), *BnaA10g04170D,* (Trehalose-phosphatase/synthase 7) on A10; *BnaC03g00620D* (Amino acid transporter, transmembrane), Winged helix-turn-helix transcription repressor *BnaC03g06370D*, *BnaC03g06560D* (Exoribonuclease), *BnaC06g36530D* (Knottin), *BnaC06g36620D* (Glycine cleavage H-protein), *BnaC06g36640D* (Thioredoxin-like fold) and *BnaC06g36720D* (EMBRYO DEFECTIVE 1793) on C06; and *BnaC09g48310D* (Multicopper oxidase, type 1) on C09 (Table S13). These putative candidate DEGs require further validation for their role in modulating water use efficiency/drought tolerance via functional gene analysis studies.

## 3. Discussion

This study investigated the genetic variation and responses to water-deficit conditions in morphological, physiological and transcriptional regulation attributes in contrasting DH lines derived from an F_1_ cross between BC1329 (maternal parent) and BC9102 (paternal parent) that show segregation for a range of plant development, physiological and productivity traits involved in drought resistance mechanisms [17,22,27].

### 3.1. Trait Plasticity to Water Deficit (Response)

We found that most of the accessions (23/24) lost at least 49% of the yield under WD conditions (Table S3b), suggesting that drought tolerance does not prevail in the germplasm evaluated, as the accessions fail to maintain crop productivity under WD conditions and do not fit with the criteria proposed by Liu et al. [33]. Under WD conditions, a significant variation in the productivity traits, such as seed yield and HI (Table S3b), could have occurred due to genotypic responses affecting plant development (shoot biomass/plant height), physiological (leaf thickness/leaf water content, leaf specific weight and Δ^13^C) parameters through the alteration of photosynthetic assimilation and remobilisation. Water is essential for various plant development, physiological, metabolic and reproduction-related activities. However, to cope with WD conditions, plants deploy different mechanisms for their survival and reproductive success. This was evident from trait plasticity (Table 2 and Table S3); different genotypes responded variably, including in their gene expression profiles in the parental lines of the DH population, especially under WD conditions, suggesting that genetically controlled variation for drought adaptation strategies, rather than drought tolerance, exists among *B. napus* lines.

Our data showed that phenotypic plasticity across water treatments is also trait-dependent and governed by genotype x environment; response to water deficit was smaller in the days-to-flower (up to 1.1-fold) trait than in seed yield (up to 17-fold, Table S3b). Our results revealed that water-deficit treatment does not accelerate flowering, a trait associated with a drought escape strategy [34,35] in test accessions, in contrast to findings made earlier in *B. rapa* [36]. Furthermore, early-flowering lines yield higher than late-flowering accessions, even in well-watered conditions (Figure 2), suggesting that early flowering does not pose any yield penalty. These observations are consistent with the findings made in Arabidopsis and canola [19,20,21,22,37,38].

### 3.2. Potential Proxy Seedling Traits for Selection of Genotypes with Improved Seed Yield

The selection of canola varieties with different phenology and herbicide chemistries for high seed yield in optimal environments has been very successful and has ushered in canola production worldwide. However, only a few traits, such as early- to mid-flowering time, early vigour and early- to mid-maturity time, have been selected for targeting canola varieties under water-limited conditions. For example, a canola variety, CB-Telfer, has been selected for a water-limited environment in Australia; this variety showed early flowering and high yield compared to longer-season spring and winter hybrid cultivars, especially under WD conditions (Table S3). We attempted to prioritise seedling traits at the vegetative stage, which can be selected in a glasshouse/field as proxies for improved seed yield, especially under WD conditions, based on correlation with seed yield—the outcome of plant and environment interactions in a particular growing environment. We found that shoot/plant biomass, leaf specific weight, leaf water content and Δ^13^C can be used as proxies to select genotypes having high seed yield in breeding programs at the seedling stage, as they had moderate to high (*r* = 0.44 to 0.83) correlation with seed yield (Figure 2). High trait correlation could be due to the tight genetic linkage of loci affecting different traits or the similar genetic–physiological module network driving trait variation. High-yielding accessions (with early flowering, increased plant height, high leaf water content, early plant biomass accumulation, high CO_2_ assimilation, low stomatal conductance and high Δ^13^C) tend to deploy drought escape mechanisms for reproductive success rather than invest available resources (e.g., photoassimilates) to produce extensive root systems that can support high yield under favourable conditions. LWC showed a positive correlation (*r* = 0.76) with seed yield, consistent with previous observations [39], supporting that hydrated and cooler canopies have higher yields than hotter ones [40]. Our results suggested that genotypes having thinner leaves with low LSW are likely to flower early and yield higher, irrespective of water treatment. In contrast, genotypes with thicker leaves (high LSW), late flowering, low LWC and low Δ^13^C fail to yield higher under both water-deficit and heat-stress and WW conditions, as observed herein (Figure 2). Nevertheless, these lines have genetic and physiological mechanisms for survival in WD conditions. Similar findings were made in glasshouse and field experiments, where a positive relationship between Δ^13^C and seed yield [17,22] is contrary to the TE concept proposed by Farquhar et al. [9]. Further research is required to test those accessions under temperature-controlled environments and whether such environments are conducive to teasing apart genetic variation and underlying genes for improved transpiration efficiency and water productivity.

Earlier studies have shown that root attributes, such as root angle, increased xylem diameter (decreased axial resistance), deep root systems, increased root length and increased root hair density contributing to higher root biomass, are important for resilience to drought and improving water use efficiency/productivity [41,42], with some exceptions [43]. These attributes enable plants to access water and nutrients from the subsoil and maintain internal water status for physiological and metabolic activities. In this study, we revealed a negative correlation between root traits (root length and root biomass) and shoot biomass, *i*WUE and Δ^13^C, plant height, seed yield and harvest index across water treatments, suggesting a trade-off between resource allocation for root development, drought avoidance and productivity traits.

### 3.3. DEGs and Their Network in Response to Water Deficit

The morphological and physiological responses to WD could be attributed to cellular and metabolic processes, as several DEGs are involved in regulating plant response to WD signalling and transduction pathways to optimise plant growth and water use. WD upregulated stress-responsive genes, including ABA-dependent and independent pathways such as CAP160, ABA receptors (*PYR1*), SnF1-related KINASE2, *DSK2*, *PP2C*; transcription factors (*RESPONSE TO DESICATION 26*, WRKY46/54/70 and AP2/ERF/TINY; *EXO70A3*, *PIN-FORMED 4* and *NCED3* [44,45] were identified. Hormonal signalling perceived under drought stress, such as the accumulation of ABA, is well established in plant growth regulation and plays a vital role in water use efficiency via auxin, brassinosteroids and stomatal closure. Genetic transformation studies show the candidacy of *PYR*, *SnRK2* and *PP2C* genes in regulating WUE [46,47]. We also identified the *BnaA10g24990D* gene, encoding a member of the *PYR* (pyrabactin resistance; *PYL(PYR1-like)*/RCAR (regulatory components of ABA receptor) family of proteins that function as abscisic acid sensors and mediate ABA-dependent regulation of protein phosphatase 2Cs ABI1 and ABI2 (Brassinosteroid signalling regulators) involved in ABA-mediated WUE [48]. Senescence-associated genes, *BnaA08g14510D* and *BnaC02g43460D* (Table S8), that showed differential gene expression are involved in ethylene signalling pathways. We identified several genes involved in cell wall biosynthesis (BnaA08g14630D-pectin, cellulose), abiotic stress tolerance transporter (*BnaA09g42040D*, *BnaC02g04280D* and *BnaA08g13490D*), carbon cycle (*BnaA0817590D*, *BnaC02g43250D* and *BnaC09g46830D*), and ABC transporters (*BnaC03g0066D* and *BnaC03g38060D*) that play roles in various plant development processes such as gametogenesis, seed development, seed germination, organ formation and secondary growth [49]. QTL for Δ^13^C on chromosomes A02, A07 and A08 were repeatedly detected in three environments (FT17, FT18 and ROS17) near the SAUR genes (Table S13), which induce cell elongation and plant growth by cell wall acidification [29,50]. SAUR can interact with PPC2C.D phosphatases to regulate plant growth by cell wall acidification [50]. Overall, transcriptomic analyses revealed several potential candidate DEGs which can be further tested for their roles in modulating water use efficiency, crop productivity and trait variation through functional gene analysis.

### 3.4. Limitations

In this study, we did not apply water to test accessions on demand; instead, we applied a fixed amount of water (100% field capacity to WW and 50% field capacity to WD treatments). Therefore, we could not determine the transpiration efficiency/water productivity (seed yield per unit of water applied). Also, we experimented with pots; therefore, genetic variation for root length may not be truly reflective of the genetic potential of different accessions under field conditions. However, root biomass measurements were reliable (high *H*^2^), and they showed a negative relationship with seed yield. This study also utilised different accessions that had genotypes that varied in flowering time. Future studies should consider isogenic lines that differ for WUE/Water productivity traits and not for flowering time to identify genes associated with high yield under drought stress.

## 4. Materials and Methods

### 4.1. Plant Material

The present study utilised a total of 24 accessions of *B. napus*: two parental lines, BC1329 and BC9102, as well as 16 lines representing extremes in the leaf Δ^13^C signatures and seed yield of the doubled-haploid (DH, designated as 11-5101) population derived from a cross between BC1329 and BC9102 [17], plus six diverse check accessions of *B. napus*. Extreme accessions assessed in our previous studies conducted in field and rainout shelter environments were selected based on their overall performance indices, determined using the Factor-Analytic approach [21,51]. Six diverse check accessions of *B. napus* represented Australian hybrid (Hyola970CL) and open-pollinated (AGC214, CB-Telfer, SturtTT and RocketCL), as well as one accession of fodder swede (NZ Sensation).

### 4.2. Experimental Design

Three experiments were conducted to (i) detail phenotypic variation in vegetative water use efficiency under contrasting water regimes (well-watered and water-deficit); (ii) detail phenotypic correlations among traits involved in vegetative water use efficiency and seed yield water use efficiency under well-watered and water-deficit conditions; and (iii) investigate transcriptome changes between parental lines in contrasting water regimes and anchor differentially expressed genes underlying QTL for plasticity in water use (Table 1).

#### Experiment 1–2

All 24 accessions were screened in six replicates under two water regimes: wet (100% field capacity) and dry (water deficit, 50% field capacity). Accessions were laid out on six benches as shown in Table S1. The pots were arranged in three adjacent blocks (bays) of 24 rows by 4 ranges of rectangular arrays, where each block contained two replicates of each line, each with two water regimes side-by-side, using a split-plot design with lines allocated to main plots and water regimes allocated randomly to the subplots as described previously [17].

Twenty-four seedlings per accession were raised in plastic trays (7 × 8 cells) containing seedling potting mix (Searles, https://www.searlesgardening.com.au/products (accessed on 31 August 2026)) in a glasshouse at the Wagga Wagga Agricultural Institute, Wagga Wagga, NSW, Australia (−35.050844, 147.348731). Two seedlings (3 weeks old) of equal vigour were selected and transplanted to a round clear plastic pot (12 inches in diameter) containing one kg of potting mixture (Searles, Kilcoy, QLD, Australia) and 70 g peat moss (Bunnings, Burnley, VIC, Australia). Each pot was weighed for its mass and saturated with water to determine its full field capacity (100%). Saturated pots were covered with a 2–3 cm thick layer of high-density polyethylene beads (HDPE) to avoid water evaporation from the soil surface, allowed to drain overnight and then weighed on an electronic scale the next day. We also included 12 pots that were randomly placed across six benches to determine the water evaporation from control pots (unplanted pots). Plants were fertilised with Thrive (NPK 25: 5: 8.8, S 4.6, Mg 0.5, Fe 0.18, B 0.005, Cu 0.005, Zn 0.004, Mo 0.001), dissolved in water as recommended by the manufacturer (Yates, Padstow, NSW, Australia, https://www.yates.com.au/product/yates-thrive/ (accessed on 31 August 2026)) fortnightly. Two sequential experiments were conducted: a short-term dry-down experiment to measure WUE/transpiration efficiency (April to June 2020, 8 weeks) and a long-term continuous experiment to measure water use and seed yield traits (April to December 2020).

For experiment 1 (short-term dry-down experiment), five-week-old plants were subjected to water treatments: WW pots were saturated at 100% field capacity (300 mL), and WD pots were watered at 50% field capacity (150 mL). Control plants were watered every 2nd or 3rd day at full capacity; water-deficit plants were left to dry out until the first wilting symptom. After 10-day periods of the short-term dry-down cycle, one random seedling was pulled out from each pot/replicate and weighed for its vegetative biomass. For experiment 2, the second plant of each accession across both treatments (24 accessions × 2 treatments × 6 reps) was grown to full maturity, and data on 13 phenotypes related to development, physiological and productivity traits were collected (Table 1).

### 4.3. Phenotypic Trait Assessments

#### 4.3.1. Agronomic and Water Use Efficiency Traits

Flowering time (days to flowering) was assessed daily and recorded when the plant had at least one open flower. Plant height (cm) was evaluated from the soil surface to the top of the inflorescence of the main stem at physiological maturity (when pod colour changed from green to yellowish, BBCH scale 80–85). At the end of the experiment, the above-soil shoot biomass was measured from each accession; biomass cuts were made from the hypocotyl/root junction and subsequently weighed on a digital balance to two decimal places. Shoot biomass at maturity was expressed in g plant^−1^ on a fresh (FW) and dry weight (DW) basis. Samples were dried in a dehydrator at 70 °C for 96 h until they reached constant mass. All accessions were hand-harvested, and the seed was cleaned with Kimseed (https://kimseed.com.au (accessed on 31 August 2026), Wangara, WA, Australia) and then weighed in the laboratory. Seed yield was expressed in g plant^−1^. The harvest index was calculated as the ratio between seed yield and total above-ground shoot biomass (dry) at harvest time. Root weight and root length measurements were carried out once the plants were measured for harvest index (within 2–3 days of harvest). The pots were thoroughly saturated with tap water in a large tub, and the roots were washed gently under running water to remove dirt and potting substrates. Roots were air-dried overnight in the shade, and then the fresh weight and length of the primary root were measured. Subsequently, root samples were placed in paper bags, dried in a dehydrator at 70 °C for 72 h, and weighed to estimate their mass. Two winter-type accessions, Hyola970CL and NZ Sensations, flowered very late, and data on flowering time and seed yield were not considered for correlation analysis.

#### 4.3.2. Physiological Trait Measurements

CO_2_ assimilation rates (*A*), stomatal conductance (*g_s_*) and intrinsic water use efficiency (*i*WUE, A/*g_s_*) were measured with a LI-COR machine (LI-COR Inc., Lincoln, NE, USA) as described previously [21]. A total of 96 samples from 2 replicates of WW and WD were measured for gas exchange per day. For gas exchange measurements, the 5th fully expanded leaf (from the crown) was selected and tagged. Gas exchange parameters were used as described in a previous study [21]. At the end of gas exchange measurements, the 5th leaf from each accession was measured for its fresh weight. Then, two discs (9.08 cm^2^) were taken from the same leaf with a Chef’s cake cutter (Kitchen, Wetherill Park, NSW, Australia) and weighed for their fresh weights. Leaf discs (leaf-specific weight, LSW) were phenotyped for their dry weights. The water content of the leaf disc (LWC, dry weight basis) was measured as the ratio of the difference between the dry and fresh leaf discs (taken from 5th leaf). To assess variation in Δ^13^C—the proxy of intercellular (*C_i_*) and the atmospheric (*C_a_*) partial pressure of CO_2_ for integral measure of WUE [52] under contrasting water regimes—the stable carbon isotope composition was determined from the remainder of the (5th) healthy leaf (otherwise, we used the 6th leaf if the 5th leaf was senescing/necrotic) (at first flower initiation). The same leaf was used for leaf thickness and repeated measures for gas exchange parameters. Leaf samples from each accession were placed in a paper bag, dried at 70 °C for 48 h in a fan-forced dehydrator and subsequently processed as described previously [21]. Intrinsic water use efficiency (*i*WUE) was expressed as the ratio between photosynthesis and stomatal conductance.

### 4.4. Statistical Analysis

All statistical analyses were conducted using software R version 4.4.2 [53]. Various phototropic and gravitropic measurements were analysed independently to determine their predicted mean with respect to 24 genotypes and the treatment (wet and dry). These predicted means for both cases of dry and wet were individually used to compute the correlation between traits, which are displayed in the heatmap for both cases. The predicted mean with 95% confidence intervals for selected traits is graphically presented. To determine the predicted means and satisfy the initial design, the linear mixed-effects model for each trait was used. Treatment was included in fixed effects, and genotypes with treatment and replicates (combinations of bay and bench) were considered as random effects. To accommodate the spatial row and column correlation within replicates, the separate autoregressive scaled AR(1) × AR(1) variance error structure was used. The package ASReml—R version 4.2.0 [54] was used for the analysis. Outliers [55] were not considered for the analysis. A Shapiro–Wilk test was used to examine the data for normality. If the data failed to satisfy the normality assumption, either the natural logarithm or square root transformation was considered, and the back-transformed [56] results were reported. Accuracy/reliability was estimated as described by Cullis et al. [57].

### 4.5. RNA Isolation, Library Preparation and Sequencing

Leaf samples from BC1329 (parent 1, P1) and BC9102 (parent 2, P2) were harvested from 12 samples (2 genotypes × 3 replicates × 2 regimes: control (well watered, WW, wet,) and water deficit (WD, dry), snap-frozen in liquid nitrogen at the time of sampling and then sent on dry ice to AGRF (Australian Genome Research Facility) for mRNA sequencing. Details of RNA sequencing are provided in the supplementary information (method 1) of our previous study [17]. The mRNA was enriched using magnetic oligo dT beads, and sequencing was performed on an Illumina NovaSeq platform, producing 12 FASTQ files. The data discussed in this publication have been deposited in the NCBI Sequence Read Archive (SRA) as PRJNA743730.

### 4.6. Read Mapping and Differential Expression Analysis

Raw data were subjected to quality control using FastQC version 0.11.2 (http://www.bioinformatics.babraham.ac.uk/projects/fastqc/ (accessed on 31 August 2026)) and screened for the presence of adapter bases and over-represented sequences. The cleaned sequence reads were then aligned against the *Brassica napus* cv. Darmor-*bzh* (*B. napus*) reference genome version 4.1 (https://bnaomics.ocri-genomics.net/analysis/8 (accessed on 31 August 2026)) using STAR aligner (v2.5.3a) (https://github.com/alexdobin/STAR/blob/master/doc/STARmanual.pdf (accessed on 31 August 2026)). The resulting BAM files were visualised using IGV (http://www.broadinstitute.org/igv/ (accessed on 31 August 2026)). For differential expression analysis, a metadata file was created containing complete information for the BAM files of all 12 samples. In addition, this metadata table also contained information about the genotype and treatment for each sample. Four experimental groups were created based on the genotype and treatment applied to the samples: P1_wet, P1_dry, P2_wet and P2_dry. Next, the counts of reads mapping to each known gene were summarised at the gene level using the featureCounts v1.5.3 utility of the R subread package (http://subread.sourceforge.net/ (accessed on 31 August 2026)). edgeR (v3.30.3) was used to perform differential expression analysis (https://bioconductor.org/packages/release/bioc/html/edgeR.html (accessed on 31 August 2026)) using R 4.0.3. A DGEList object was created from the count matrix using the edgeR package, and the calcNormFactors function was used to calculate normalisation factors for scaling the raw library sizes in the count matrix [58]. Counts of aligned reads were converted to counts per million (CPM). A gene-filter approach was then used to remove genes that were not expressed at all. A negative binomial generalised linear model (GLM) was fitted for each gene using a factorial design that included genotype (P1/BC1329 and P2/BC9102), treatment (Wet and Dry) and the genotype × treatment interaction.

Three biological replicates were included for each genotype × treatment combination (P1_Wet, P1_Dry, P2_Wet and P2_Dry). Differential expression was assessed using the following contrasts: Water-deficit DEGs in P1 = P1_Wet − P1_Dry and Water-deficit DEGs in P2 = P2_Wet − P2_Dry; DEGs with genotype effect = (P1_Wet + P1_Dry) − (P2_Wet + P2_Dry); DEGs with treatment effect = (P1_Dry + P2_Dry) − (P1_Wet + P2_Wet) and DEGs with Genotype × treatment interaction = (P1_Wet − P1_Dry) − (P2_Wet − P2_Dry). The differentially expressed genes (DEGs) were considered significant if they showed a log_2_FC change > 2.0 and a false discovery rate (FDR, *p*-values < 0.05).

### 4.7. Functional Annotation, Gene Ontology (GO) Enrichment and KEGG Pathway Analysis

*B. napus* protein sequences were downloaded from https://bnaomics.ocri-genomics.net/analysis/8. BLASTP (https://blast.ncbi.nlm.nih.gov/Blast.cgi?PAGE=Protein, accessed on 31 August 2026) searches of *Brassica* protein sequences were performed against *Arabidopsis thaliana* protein sequences with an e-value cut-off of 10^−15^. Protein sequences for *Arabidopsis* were downloaded from https://www.arabidopsis.org/download_files/Proteins/TAIR10_protein_lists/TAIR10_pep_20101214 (accessed on 31 August 2026). Reverse BLASTP searches were also performed, using *Arabidopsis* protein sequences as queries against *Brassica* protein sequences. BLASTP and reverse BLASTP results were inspected for their top hits, and subsequently, putative annotations were added to the *Brassica* proteins. These *Arabidopsis* annotations were then added to all DEGs. The DEGs were subjected to GO and pathway analyses using the gene list analysis tool from PANTHER (http://go.pantherdb.org/tools/compareToRefList.jsp (accessed on 31 August 2026)) by choosing *Brassica napus* as the organism and statistical enrichment test for the analysis. The Statistical Overrepresentation Test was implemented in PANTHER, and *p*-values were adjusted for multiple testing using the Benjamini–Hochberg false discovery rate (FDR) correction. Terms with an adjusted FDR < 0.05 were considered significantly enriched. Gene-to-function assignments were based on the *B. napus* annotations and GO classifications available within the PANTHER database. *Arabidopsis* genes annotated with various WUE-related terms (water deprivation) were retrieved from the TAIR 10 database (https://www.arabidopsis.org/ (accessed on 31 August 2026)), and subsequent annotations were added to the DEG lists.

### 4.8. DEGs in the QTL Regions and Their Association with Productivity Traits

To identify the potential candidate genes underlying adaptive and productivity traits (shoot biomass, flowering time, Δ^13^C, chlorophyll content (SPAD) and seed yield), we identified DEGs that are located within 100 kb up- and downstream of the QTL intervals [17,22]. The physical position of markers in the QTL intervals was obtained from sequence alignments of marker sequences with the reference Darmor-*bzh* genome.

## 5. Conclusions

This study investigated the impact of water deficit on *B. napus* accessions, with a significant reduction in plant growth and performance, although the flowering time was not affected. Our results showed that WD can cause a total failure of the crop (with 100% yield loss), particularly in the late-flowering accessions. RNA-seq analysis revealed a significant response in gene expression, including upregulation and downregulation of genes associated with water deficit. This study provides valuable insights into morphological and transcriptional responses to WD and potential differentially expressed gene targets, especially in the vicinity of QTL associated with traits involved in water use efficiency and drought avoidance and escape mechanisms. In addition, this study provides genetic resources for advancing our knowledge of breeding improved canola varieties under water-deficit conditions.

## Figures and Tables

**Table 1:** Description of experiments conducted to determine responses to two contrasting water treatments: wet (100% field capacity) and dry (water deficit, 50% field capacity) in agronomic and physiological attributes under glasshouse conditions. NA: not applicable.

Experiment	Objective	Materials	Phenotypic Traits Phenotyped
1: Short-time, dry-down drought experiment	To detail phenotypic variation in vegetative water use efficiency under contrasting water regimes (well-watered and water-deficit)	24 lines of *B. napus*: two parental lines, BC1329 and BC9102, 16 DH from BC1329/BC9102, plus six diverse check *B. napus* accessions	Shoot biomass
2: Long-term progressive drought experiment	To detail phenotypic correlations among traits involved in vegetative water use efficiency and yield water use efficiency under well-watered and water-deficit conditions	Same as in Experiment 1	Plant development (flowering time: DTF, plant height: PH, leaf specific weight: LSW, root weight, length of the longest root, and total plant weight (fresh and dry weight) at physiological maturity Physiological traits (*A_g_*; *g_sw_*; intrinsic water use efficiency: iWUE; Δ13; and leaf water content) Productivity (seed yield: SY, and harvest index: HI at the whole-plant level, defined as seed yield per unit water transpired), in two water regimes: Wet and Dry
3	Investigate transcriptome changes between parental lines in contrasting water regimes and anchor differentially expressed genes underlying QTL for plasticity in water use.	Parental lines, BC1329 and BC9102	NA

**Table 2:** Various traits of the parental lines and selected 16 DH lines of the 11-5101 population plus six controls (Hyola970CL, AGC214, CB-Telfer, SturtTT, RocketCL and NZ Sensation). WET: well-watered (Control); DRY: water deficit; *H*^2^: repeatability; *A_g_*: CO_2_ assimilation rate; *g_sw_*: conductance; *i*WUE: intrinsic water use efficiency; LWC: leaf water content; DTF: days to first flower; HI: harvest index.

	BC1329		BC9102		Check	Lines	DH	Lines	All	Lines
	Predicted	Range	Predicted	Range	Predicted	Range	Predicted	Range	*H* ^2^	
	WET	DRY	WET	DRY	WET	DRY	WET	DRY	WET	DRY
Yield (g)	7.21	2.82	12.41	4.57	0.00–9.86	0.00–4.61	0.41–9.53	0.00–4.41	0.98	0.93
Biomass (g)	41.96	18.21	37.29	16.63	13.84–46.40	12.1–23.0	19.02–33.72	9.5–17.16	0.97	0.94
HI	0.17	0.13	0.32	0.24	0.15–0.34	0.06–0.36	0.03–0.35	0.00–0.31	0.90	0.92
DTF (days)	74.04	76.03	70.83	72.01	62.69–91.76	61.85–98.27	62.43–121.11	61.52–134.8	0.90	0.90
Height (cm)	105.58	66.4	102.9	75.03	12.25–91.47	7.62–65.26	69.32–99.62	17.01–68.95	0.96	0.94
Dry Leaf Disc Weight (g)	0.05	0.06	0.04	0.05	0.04–0.07	0.05–0.07	0.04–0.07	0.05–0.07	0.90	0.89
LWC	8.31	6.88	9.59	8.27	6.35–11.77	5.05–10.39	7.36–10.6	5.45–8.88	0.88	0.91
Root Weight (Dry, g)	8.12	2.32	2.91	1.62	1.87–40.65	1.00–8.93	1.94–8.31	1.12–2.95	0.96	0.94
Root Length (mm)	309.01	295.03	272.64	273.88	280.02–347.54	277.16–318.28	253.19–322.42	261.05–302.76	0.64	0.63
Plant Dry Weight (g)	1.64	1.15	1.5	1.07	1.01–1.95	0.79–1.33	0.42–1.73	0.45–1.2	0.85	0.85
Plant Fresh Weight (g)	25.07	13.34	26.05	13.7	17.56–28.97	10.89–14.63	8.15–27.03	7.81–13.99	0.90	0.90
*A_g_* (µmol CO_2)_	4.6	4.54	4.38	3.81	2.76–5.54	3.53–4.88	0.54–6.17	2.3–5.69	0.73	0.55
*g_sw_* (mol H_2_O) m^−2^s^−1^)	0.03	0.02	0.04	0.02	0.01–0.04	0.02–0.03	0.01–0.05	0.02–0.04	0.78	0.40
*i*WUE	250.32	227.52	121.86	177.3	104.23–559.36	138.49–352.33	8.45–358.35	24.87–261.25	0.86	0.76
Δ^13^ (‰)	22.77	s21.67	23.14	s22.15	21.75–24.48	20.37–23.86	21.97–23.42	20.65–22.5	0.83	0.83

## Data Availability

The raw sequence data reported in this paper have been deposited in the National Centre for Biotechnology Information Sequence Read Archive (Accession no. PRJNA743730). The request for material should be addressed to Harsh Raman.

## Supplementary Materials

The following supporting information can be downloaded at: https://www.mdpi.com/article/10.3390/ijms27177967/s1.
