# Supplementary material for: Morphological, Physiological and Transcriptomic Changes in Response to Water Deficit Stress in Brassica napus L
Source: Int J Mol Sci. 2026 Sep 7;27(17):7967. doi: 10.3390/ijms27177967 (PMC13565986; doi:10.3390/ijms27177967)
Supplement: Supplementary file 1 [file ijms-27-07967-s001.zip › Figure S1.pdf]

Figure S1.

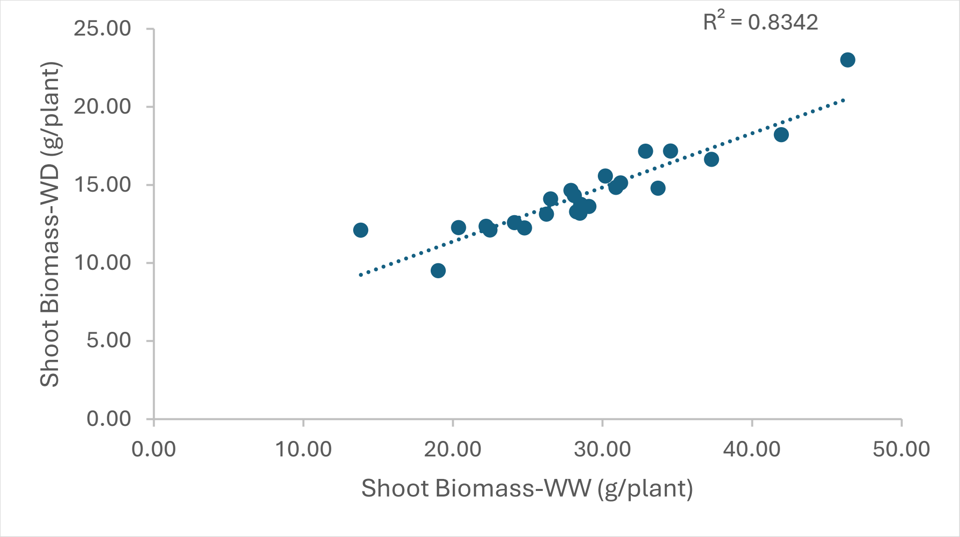

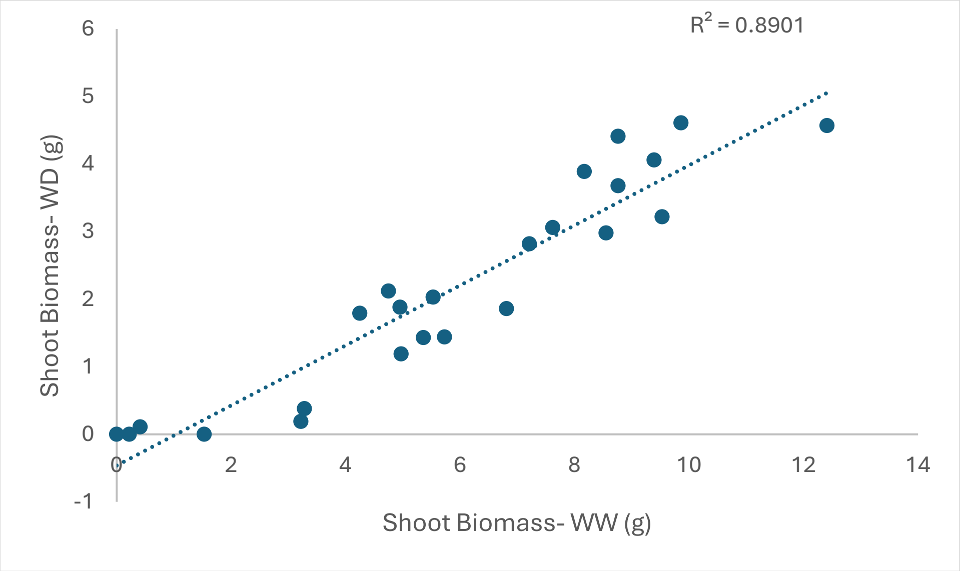

Figure S1: This multi-dimensional scaling (MDS) plot delineates distances between samples corresponding to the leading biological coefficient of variation (BCV). Samples are designated as P1D1- P1_dry_replicate 1, P1D2 – P1_dry_replicate 2, P1D3 – P1_dry_replicate 3, P1W1 – P1_wet_replicate 1, P1W2 – P1_wet_replicate 2, and P1W3 – P1_wet_replicate 3. All biological replicates of a given sample tend to cluster together, while samples from different experimental groups form separate clusters. Dimension 1 separates samples from the two parental lines, and dimension 2 separates

samples derived from the WW and WD treatments. The plot also suggests that the variability between P1 and P2 samples is larger than that between wet and dry samples of the same parental line.
