# Supplementary material for: Morphological, Physiological and Transcriptomic Changes in Response to Water Deficit Stress in Brassica napus L
Source: Int J Mol Sci. 2026 Sep 7;27(17):7967. doi: 10.3390/ijms27177967 (PMC13565986; doi:10.3390/ijms27177967)
Supplement: Supplementary file 1 [file ijms-27-07967-s001.zip › Figure S2.pdf]

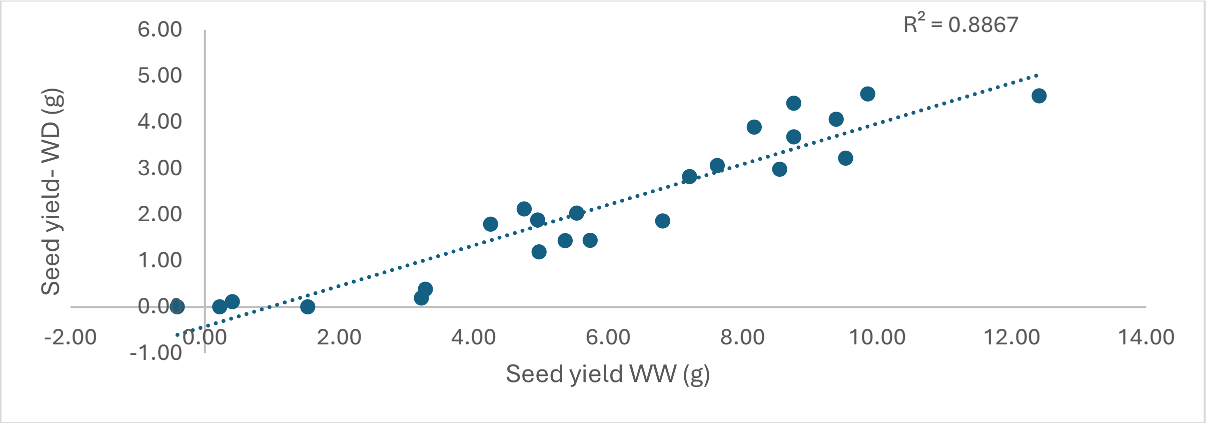

Figure S2: Relationship between seed yield in well-watered (WW) and water-deficient (WD) conditions in 24 accessions of canola.
