# Supplementary material for: Morphological, Physiological and Transcriptomic Changes in Response to Water Deficit Stress in Brassica napus L
Source: Int J Mol Sci. 2026 Sep 7;27(17):7967. doi: 10.3390/ijms27177967 (PMC13565986; doi:10.3390/ijms27177967)
Supplement: Supplementary file 1 [file ijms-27-07967-s001.zip › Figure S3-rank.pdf]

	Accession			Accession			
Rank		Seed yield (W	**Rank changed from WW t**		Seed yield (WD, g)		
**1**	P2-5101	12.41	**2**	Telfer	4.61		
**2**	Telfer	9.86	**1**	P2-5101	4.57		
**3**	06.5101.556	9.53	**5**	06.5101.137	4.41		
**4**	06.5101.026	9.39	**4**	06.5101.026	4.06		
**5**	06.5101.137	8.76	**8**	06.5101.014	3.89		
**6**	06.5101.643	8.76	**6**	06.5101.643	3.68		
**7**	AGC214	8.55	**3**	06.5101.556	3.22		
**8**	06.5101.014	8.17	**9**	Rock	3.06		
**9**	Rock	7.62	**7**	AGC214	2.98		
**10**	P1-5101	7.21	**10**	P1-5101	2.82		
**11**	06.5101.657	6.81	**17**	Sturtt	2.12		
**12**	06.5101.512	5.73	**13**	06.5101.501	2.03		
**13**	06.5101.501	5.53	**16**	06.5101.226	1.88		
**14**	06.5101.063	5.36	**11**	06.5101.657	1.86		
**15**	06.5101.413	4.97	**18**	06.5101.157	1.79		
**16**	06.5101.226	4.95	**12**	06.5101.512	1.44		
**17**	Sturtt	4.75	**14**	06.5101.063	1.43		
**18**	06.5101.157	4.25	**15**	06.5101.413	1.19		
**19**	06.5101.018	3.28	**19**	06.5101.018	0.38		
**20**	06.5101.088	3.22	**20**	06.5101.088	0.19		
**21**	06.5101.306	1.53	**22**	06.5101.009	0.11		
**22**	06.5101.009	0.41	**24**	NZ Sensation-T	0		
**23**	Hyola970CL	0.22	**23**	Hyola970CL	0		
**24**	NZ Sensation-Tu	-0.31	**21**	06.5101.306	0		

Figure S3: change in ranking
