# Supplementary material for: Morphological, Physiological and Transcriptomic Changes in Response to Water Deficit Stress in Brassica napus L
Source: Int J Mol Sci. 2026 Sep 7;27(17):7967. doi: 10.3390/ijms27177967 (PMC13565986; doi:10.3390/ijms27177967)
Supplement: Supplementary file 1 [file ijms-27-07967-s001.zip › Figure S4-PCO.pdf]

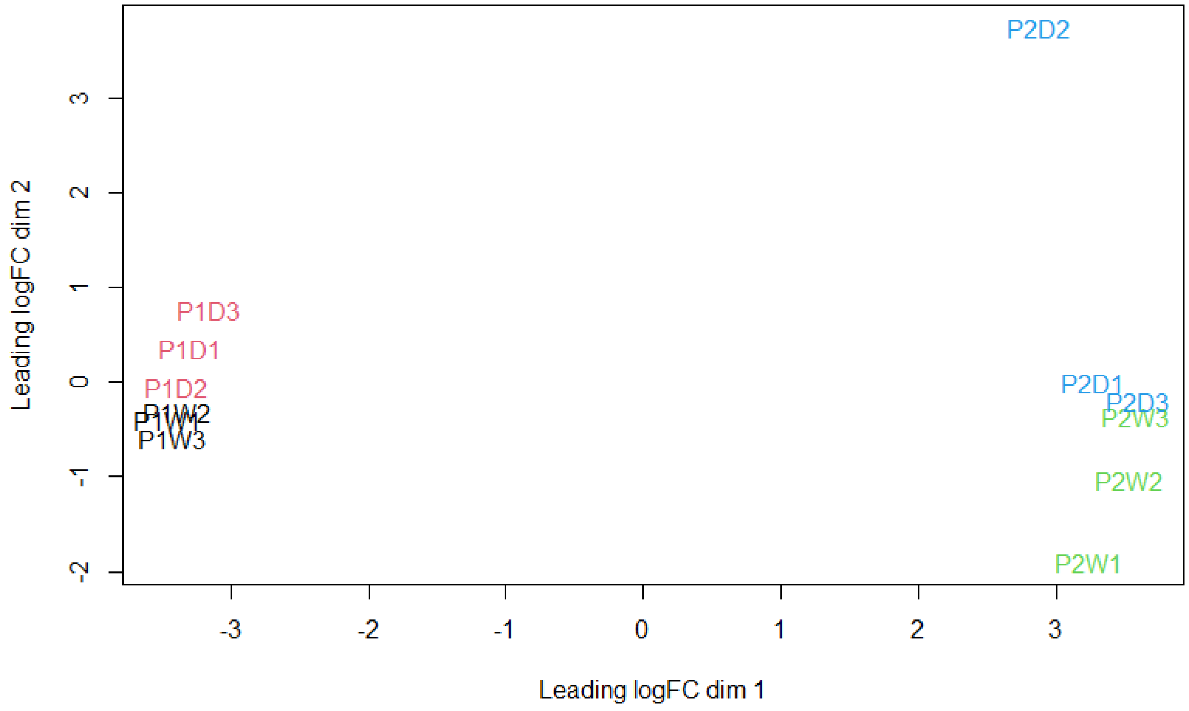

Figure S4. MDS plot showing distances between samples corresponding to leading biological coefficient of variation (BCV). All biological replicates of samples tend to cluster together, while samples from different groups form separate clusters. The plot also suggests variability between P1 and P2 samples are larger than the variability between P! wet and dry samples and P2 wet and dry samples.
